# Bacterial surface colonization, preferential attachment and fitness under periodic stress

**DOI:** 10.1371/journal.pcbi.1006815

**Published:** 2019-03-05

**Authors:** Maor Grinberg, Tomer Orevi, Nadav Kashtan

**Affiliations:** The department of Plant Pathology and Microbiology, The Robert H Smith Faculty of Agriculture, Food and Environment, The Hebrew University of Jerusalem, Rehovot, Israel; UNITED STATES

## Abstract

Early bacterial surface colonization is not a random process wherein cells arbitrarily attach to surfaces and grow; but rather, attachment events, movement and cellular interactions induce non-random spatial organization. We have only begun to understand how the apparent self-organization affects the fitness of the population. A key factor contributing to fitness is the tradeoff between solitary-planktonic and aggregated surface-attached biofilm lifestyles. Though planktonic cells typically grow faster, bacteria in aggregates are more resistant to stress such as desiccation, antibiotics and predation. Here we ask if and to what extent informed surface-attachments improve fitness during early surface colonization under periodic stress conditions. We use an individual-based modeling approach to simulate foraging planktonic cells colonizing a surface under alternating wet-dry cycles. Such cycles are common in the largest terrestrial microbial habitats–soil, roots, and leaf surfaces–that are not constantly saturated with water and experience daily periods of desiccation stress. We compared different surface-attachment strategies, and analyzed the emerging spatio-temporal dynamics of surface colonization and population yield as a measure of fitness. We demonstrate that a simple strategy of preferential attachment (PA), biased to dense sites, carries a large fitness advantage over any random attachment across a broad range of environmental conditions–particularly under periodic stress.

## Author summary

A vast portion of bacterial life on Earth takes place on surfaces. In many of these surfaces cells collectively organize into biofilms that are known to provide them protection from various environmental stresses. Early bacterial colonization of surfaces, prior to the development of mature biofilm, is a critical stage during which cells attempt to establish a sustainable population. It is not a random process wherein cells arbitrarily attach to surfaces and grow to form micro-colonies. Rather, surface-attachments, movement and cellular interactions take place to yield non-random organization. Using computer simulations, based on individual-based modeling, we demonstrate that simple attachment strategies, where planktonic cells preferentially attach to existing surface-attached aggregates, may confer fitness advantage over random attachment. The advantage of preferential attachment is particularly substantial under periodic stress–a common characteristic of many natural microbial habitats. This is due to a more efficient recruitment of planktonic cells that accelerates the formation of stress-protected aggregates.

## Introduction

Early bacterial surface colonization that takes place prior to the development of mature biofilm, is a critical stage during which cells attempt to establish a sustainable population [1]. There is growing evidence that this is not a random process wherein cells arbitrarily attach to surfaces and grow to form microcolonies. Relocation and detachment of cells were shown to play a major role in bacterial colonization on leaves [2, 3]. A “rich get richer” process was observed during the surface colonization of *P*. *aeruginosa*, where some aggregates (cell clusters) are enriched by biased surface movement of cells controlled by a web of secreted polysaccharide [4], and surface-attachment of planktonic cells was shown to be biased toward lower distances to previously attached cells [5]. Thus, early surface colonization seems to be a self-organized process, resulting from the behavior of individual-cells. Yet, we know very little of how such individual-cell behavior and the emergent self-organization affect the fitness of the population.

**Fig 1:**
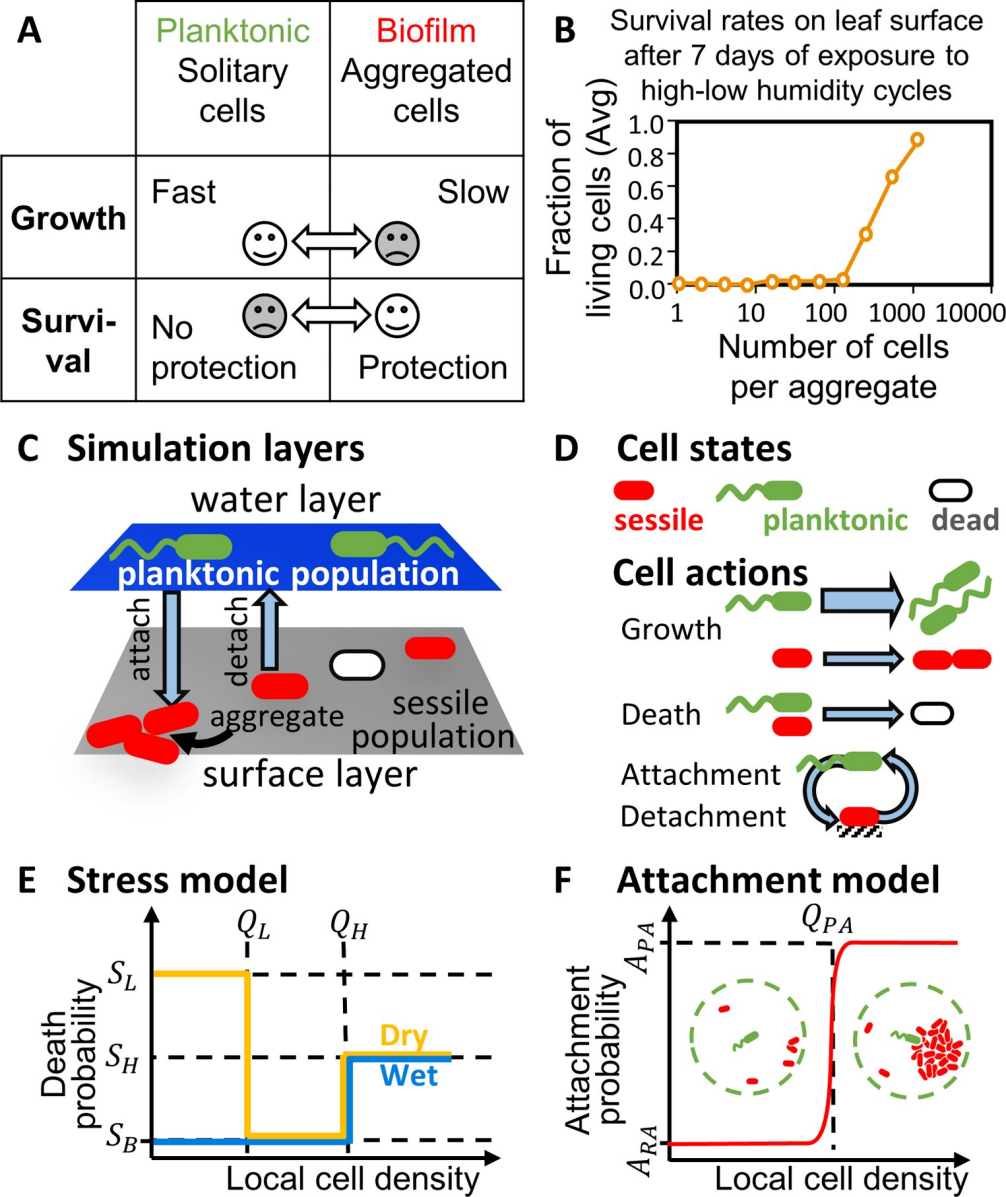
**Model description A**. The inherent tradeoff between planktonic cells and biofilms.**B**. Fraction of live bacteria cells per aggregate size on bean leaf (adapted from [19]). In essence, cells within smaller aggregates do not survive prolonged and recurring dry periods while cells in larger aggregates do. **C**. Schematic depiction of the model. The top layer is the bulk fluid, hosting the planktonic sub-population. The bottom layer is the surface, hosting the sessile sub-population. **D**. The model includes planktonic and sessile cells. At each time step, the cells may grow and divide, die due to stress, and change their state. **E.** Stress is modeled as the hourly probability of cell death, and is a function of both local cell density and the hydration state. The probability to die due to desiccation is high in aggregates below the protection threshold Q_L_. In addition, to account for overcrowding, the probability of death increases above density Q_H_. **F.** Preferential attachment is modeled as a step-like function of local cell density.

A key factor in overall population fitness is the inherent tradeoff between the planktonic and biofilm lifestyles [6–9]. Planktonic cells typically grow faster by eluding the costly production of extracellular polymeric substances (EPS) that is common in biofilms [10, 11] and by avoiding the high cell density and limited diffusion within aggregates [12]. Aggregates on the other hand, provide protection from various stresses including desiccation, antibiotics, and predation [13–18] (Fig 1A). Often, protection from stress within aggregates is collective and is a function of the size of the aggregate, with a minimal size required to achieve protection. For example, protection from desiccation on plant leaf surfaces was shown to be gained in aggregates above ~100 cells [19] (Fig 1B).

The decision of a planktonic cell to attach to the surface and change its lifestyle is a function of several factors including environmental cues, surface properties, and its physiological state [20–22]. These individual cells’ decisions play an important role in their proliferation and survival, as a consequence of the aforementioned tradeoff. Since individual-cell behavior modulates aggregate formation dynamics, it affects the fitness of the collective through self-organization. Therefore, the mechanisms governing these decisions are expected to be under strong selection [7, 8, 23, 24].

In many microbial habitats stress conditions are not continuous but vary with time, often in a periodic manner. The largest terrestrial microbial habitats–soil, plant root and leaf surfaces–are not constantly saturated with water, and experience daily recurring wetting and drying dynamics [25, 26]. Cells on plant leaf surfaces and shallow water of marine environments are periodically exposed to strong UV radiation at mid-day [27]. Bacterial grazing in the marine environment can be periodic over the diel cycle [28]. Human microbiota are likely exposed to daily variation in immune system activity [29] or in antibiotic levels [30]. Thus, periodic stress is likely very prevalent during bacterial surface colonization.

Temporal variation in stress levels often favors a division of labor between cell phenotypes, i.e. reproductive specialists and survival specialists (e.g. planktonic and biofilm cells, respectively) [9]. The tradeoff of planktonic and biofilm phenotypes has been studied in several contexts [7], including the role of life-style transitions in periodically fluctuating environments [31], and both analytic and individual-based models were used to explore the mechanisms that optimize fitness and balance the population composition [9, 32].

Because aggregate size plays a role in survival, it can be beneficial to accelerate the development of aggregates, especially when growth rate is too low to support the formation of protected aggregates in the intervals between stress periods. Since clonal growth rate is constrained by cell physiology and environmental conditions including nutrient availability, an alternative way to accelerate aggregate growth is by recruitment of other cells; either by movement of nearby, already surface-attached, cells (as observed by Zhao et. al [4]), or by attachment of foraging planktonic cells to, or near, the aggregate. We term such self-organizing mechanisms that enrich or promote aggregation Aggregate-Enrichment Mechanisms (AEMs). One such mechanism is preferential attachment (PA), which we use as a general term for attachment pattern that is biased towards attachment to existing aggregates, and thus depends on the spatial organization of sessile bacteria on the surface. A PA process is a general stochastic growth process wherein individuals join groups in a system in a “biased” non-random way. PA has been shown to be common in many real-world complex systems such as networks where new nodes are preferentially attached to nodes with high connectivity [33]. PA has been demonstrated to explain the degree distribution of scale-free networks including many biological and social networks [34].

We hypothesize that PA plays an important role in early bacterial surface colonization under environments that select for collective protection, through the self-organization induced by biased individual surface-attachment decisions. Here we ask if and to what extent PA strategies can provide fitness advantage in environments with periodic stress. We use an individual-based modeling approach [35, 36] to simulate early bacterial surface colonization under diel cycles of alternating wet and dry periods, confronting cells with periodic desiccation stress. Our simulations allowed us to compare different PA strategies with random strategies, to analyze the emerging spatio-temporal dynamics of surface colonization, and evaluate population yield as a measure of fitness.

## Results

### Individual-based model of bacterial surface colonization

To study the fitness advantage of PA under periodic stress, we developed an individual-based model of bacterial surface colonization in fluctuating hydration conditions. The model consists of two layers: the fluid and the surface inhabited by planktonic and sessile (i.e. biofilm) bacterial cells, respectively (Fig 1C). Planktonic cells are motile while sessile cells are not (except when passively moved). Nutrients are assumed to diffuse into the system from a constant concentration source. Cells grow and divide in accordance with the available nutrient levels and the cells' state, die as a function of stress level and cell-density, and may attach to the surface or detach from the surface (Fig 1D). Cells' growth parameters are typical for environmental bacteria that colonize the leaf surface [37] (Tables 1 and 2, Eq 1).

**Table 1:** Model parameters.

Parameter	Symbol	Value	Unit	Ref.
Individuals:				
Maximum growth rate (planktonic cells)	μ_max_	0.4	h^-1^	[37]
Nutrient affinity constant	K_s_	0.3	g∙m^-3^	[37]
Fraction of biomass invested in EPS production (biofilm cells)	f	0.5		[38]
Cell-division mass (mean)	M_div_	2e-12	g	[39]
Nutrient consumption per cell division	C_div_	3e-13	g	[3]
Radius of neighborhood	R	10	μm	
Maximal swimming speed of planktonic cells	V	~50	μm∙s^-1^	[40]
Domain:				
Permeation rate of nutrients into the domain		0.1	h^-1^	
Domain area		1	mm^2^	
Water volume		0.1	mm^3^	
Time step	*dt*	0.05	h	
Stress and attachment probability function parameters: (default values)				
Probability of cell death due to desiccation stress	*S*_*L*_	0.4	h^-1^	
Probability of cell death due to high cell density	*S*_*H*_	0.3	h^-1^	
Probability of cell death due to other reasons	*S*_*B*_	0.025	h^-1^	
Density threshold of desiccation stress	*Q*_*L*_	40		
Density threshold of overcrowding stress	*Q*_*H*_	75		
Attachment probability with PA	*A*_*PA*_	0.5	dt^-1^	
Attachment probability with RA	*A*_*RA*_	0.01	h^-1^	
Detachment probability	D	0.01	h^-1^	

**Table 2:** Equations and functions.

	Name	Equation	Units
(1)	Growth Kinetics	$m_{i}(t+\Delta t)=(1-f_{i})\cdot m_{i}(t)\cdot \exp(\mu_{max}\frac{c(x_{i},t)}{c(x_{i},t)+K_{s}}\Delta t)+f_{i}m_{i}(t)$ *c*(*x*,*t*): nutrient concentration *f*_*i*_: *i*-th cell’s investment in EPS	*g*
(2)	Probability of cell death	$S_{i}(x_{i},t)=\left\{ \begin{array}{cc} (1-W(t))\cdot S_{L}+S_{B} & Q(x_{i},t)<Q_{L}\\ S_{H}+S_{B} & Q(x_{i},t)\geq Q_{H}\\ S_{B} & \mathrm{otherwise} \end{array}\right.$ $W(t)=\left\{ \begin{array}{cc} 1 & \mathrm{mod}(t,24)\leq H\\ 0 & \mathrm{mod}(t,24)>H \end{array}\right.,\;H$: duration of the wet period	*h*^−1^
(3)	Attachment probability	$A_{i}(x,t)={(A}_{PA}-A_{RA})\cdot \frac{1}{{\left(\frac{Q_{PA}}{Q_{i}(x,t)}\right)}^{n}+1}+A_{RA}$	*h*^−1^
(4)	Detachment probability	*D*_*i*_(*x*,*t*) = *D*	*h*^−1^

Importantly, our model employs the tradeoff between growth and survival that is associated with the planktonic and biofilm lifestyles (Fig 1). Here, sessile cells devote part of their resources to the production of EPS and thus grow slower than planktonic cells (see Methods). The effect of stress is modeled by the hourly probability of cell death (S) that is a function of both the local cell density and the hydration conditions. S of solitary cells and cells within small aggregates is low (S_B_) during wet periods and high during the dry periods (S_L_). Cells in larger aggregates (densities >Q_L_) gain protection and thus have low death probability (S_B_) during both wet and dry periods. To account for nutrient deprivation and toxin buildup at the centers of established biofilms, S increases to S_H_ at densities higher than Q_H_ (Fig 1E, Tables 1 and 2, Eq 2).

To model attachment, we used a probability function which describes the chances of planktonic cell attachment. The attachment probability per time step (A) of a planktonic cell depends on the local cell density of surface-attached bacteria, defined as the number of sessile cells in 10μm-radius neighborhood. Cells that do not enact PA, and rely on random attachment (RA) alone, have a constant A_RA_ attachment probability. The attachment probability function for cells with PA is a step-like function, as described in Fig 1F. Other probability functions were tested, but did not show significant advantage over the step-like function (See S1 Fig). When a planktonic cell with PA encounters local sessile-cell densities above *Q*_*PA*_, it has a higher attachment probability (*A*_*PA*_ > *A*_*RA*_). Last, in our model, detachment (biofilm-to-planktonic transition) rate is constant and equals D = 0.01 [*h*^−1^] (Tables 1 and 2, Eqs 3 and 4).

**Fig 2:**
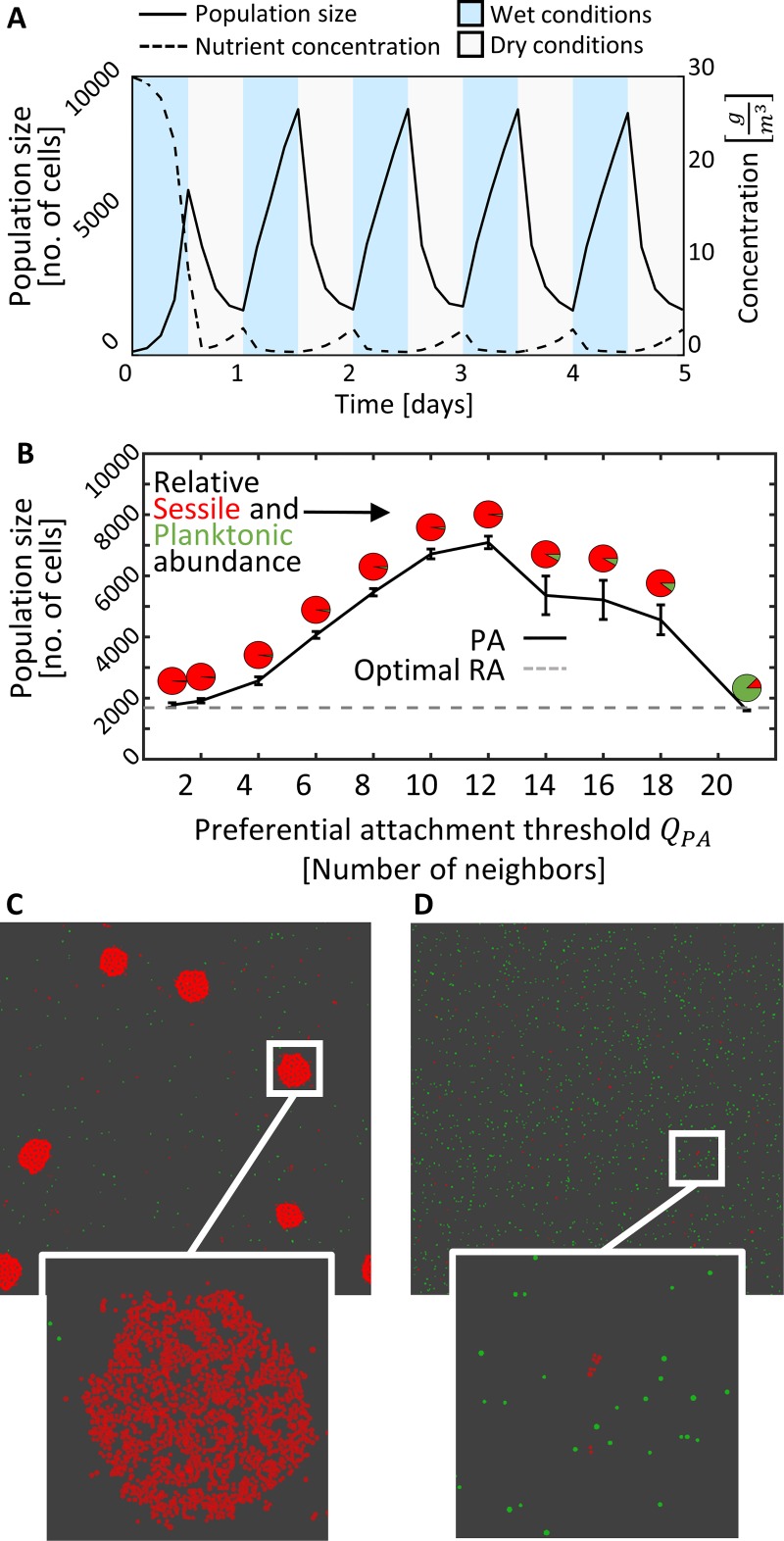
PA can confer fitness advantage and there is an optimal attachment threshold. **A.** Typical simulation dynamics of population size and nutrient concentration over five diel cycles. In general, the population size increases during the wet periods and decreases during the dry periods. Fluctuations in population size are dictated by nutrient availability, periodic stress and spatial arrangement of the cells. Dynamics presented from a simulation with RA where cells attach and detach at the same constant probability (*A*_*RA*_ = *D* = 0.01 [*h*^−1^]) **B.** Population sizes at the end of a five diel cycle simulations with different PA thresholds (Q_PA_). Each data point is mean±SE of 10 simulations. Pie diagrams represent relative abundance of planktonic and sessile cells. The dashed line represents the population size of the optimal RA simulation, i.e. RA with the highest yield among all tested A_RA_ values. **C.** Snapshot from the optimal PA strategy (Q_PA_ = 12). **D.** Snapshot from the optimal RA strategy. Snapshots C and D were taken at the end of day 5. Sessile cells are shown in red, planktonic in green.

Our simulation begins with 100 planktonic cells and an empty surface. A simulation lasts five diel cycles, with alternating 12-hour long wet and dry periods. Fitness is assessed by the final population size (population yield) after the last dry period. Typical overall population dynamics, with RA at a rate equal to the detachment rate *A*_*RA*_ = *D*, are shown in Fig 2A. After the initial growth the population oscillates periodically, in accordance with the inbound nutrient flux and the stress-induced death rate, determined by hydration conditions and the spatial organization of cells on the surface.

### Preferential attachment can improve fitness

**Fig 3:**
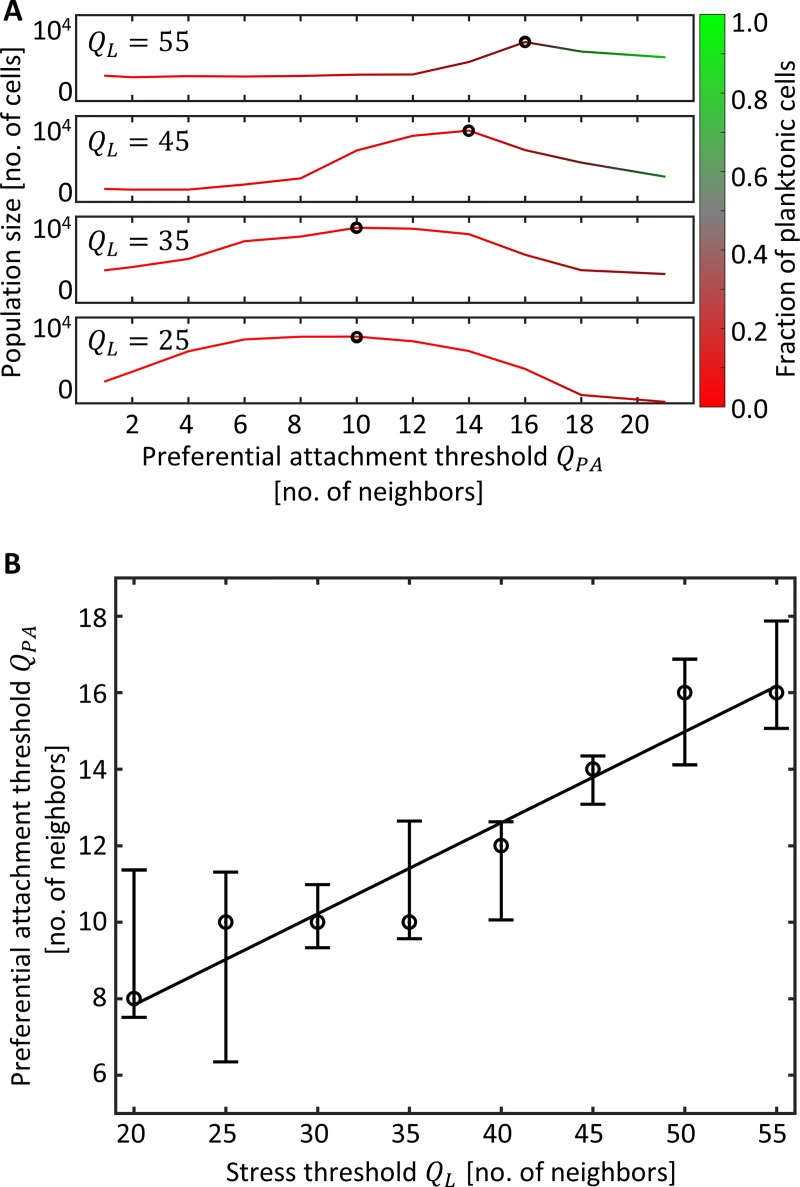
Optimal preferential attachment threshold depends on protected density threshold. **A.** Comparison of different preferential attachment thresholds (Q_PA_), corresponding to different strategies, at various conditions differing in desiccation stress density thresholds Q_L_. Each data-point is the mean of 10 simulations. Color indicates the fraction of planktonic cells from the entire population. Black circle marks the optimal strategy. **B.** Interpolated optimal attachment threshold Q_PA_ as a function of the desiccation stress density threshold Q_L_.

To study the impact of PA strategies on the dynamics of surface colonization, we compared simulations with a range of PA strategies (represented by different threshold values *Q*_*PA*_) as well as simulations with RA with a range of *A*_*RA*_ rates. We then compared the mean population size, i.e. yield, at the end of a five diel cycles simulation, as a measure for fitness. Remarkably, we find that under the simulated conditions, there are PA strategies that confer a significant fitness advantage (i.e. a higher yield) over any random attachment rate. Moreover, the optimal strategy is at an intermediate *Q*_*PA*_ value, and fine tuning of *Q*_*PA*_ has a large effect on fitness (Fig 2B). Snapshots of the final simulation state for the optimal PA and RA simulations are shown in Fig 2C and 2D. Two representative simulations are shown in S1 Video. Different Q_L_ values (size required for protection) had an impact on the value of the optimal attachment threshold (the higher the Q_L,_ the higher the optimal Q_PA_) but the overall picture remained similar (Fig 3).

### Surface colonization dynamics

**Fig 4:**
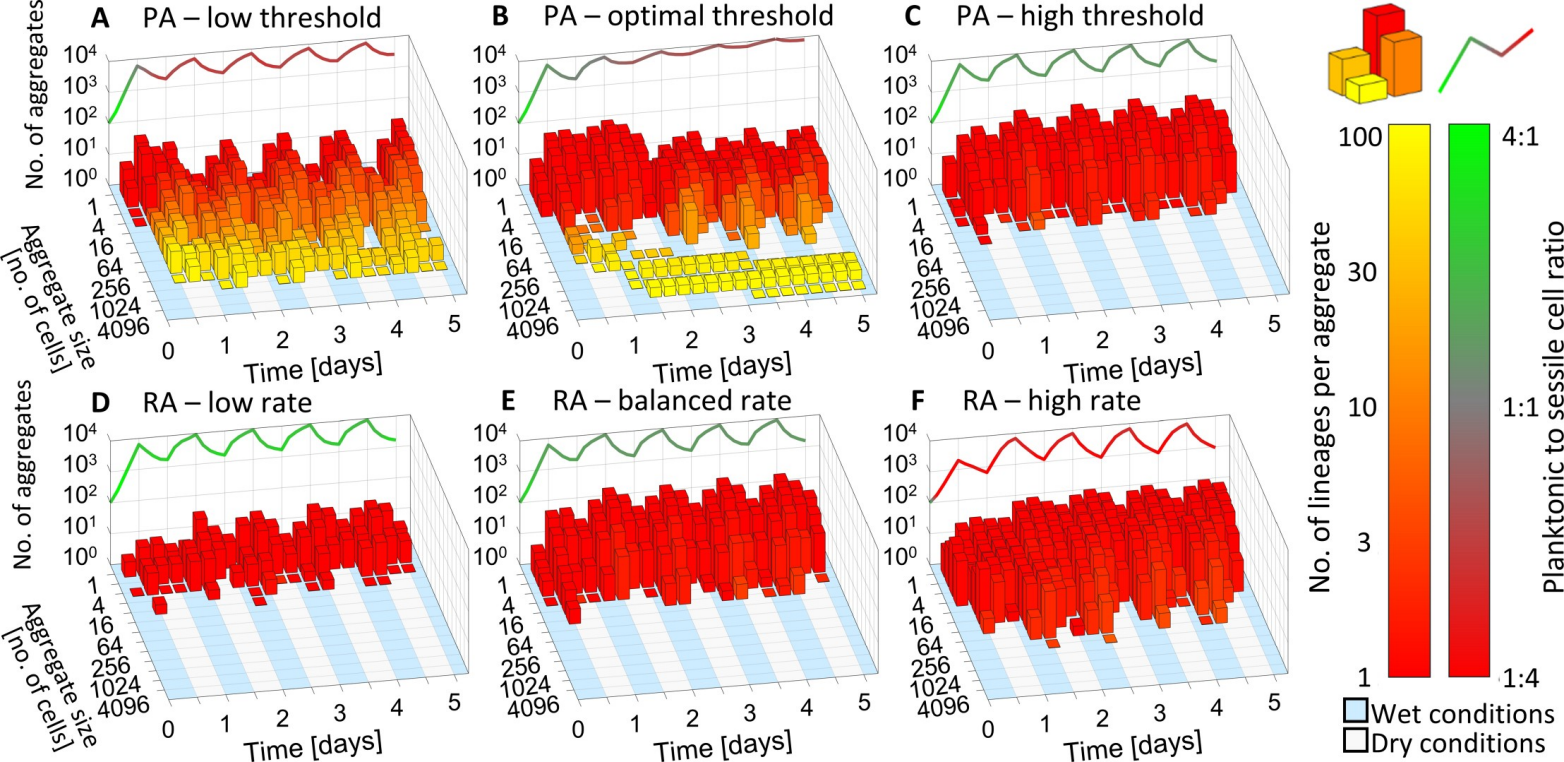
Aggregate-distribution dynamics help explain how the optimal PA strategy works. **A-F.** Aggregate-size distribution dynamics. Bar height indicates number of aggregates within size range at specific time point. Bar color indicates the average number of different linages per aggregate. The curve on top shows total population size and ratio of planktonic to sessile cell count **A.** PA with *Q*_*PA*_ = 4. **B.** PA with *Q*_*PA*_ = 12, **C.** PA with *Q*_*PA*_ = 20, **D.** RA with *A*_*RA*_ = 0.001, **E.** RA with *A*_*RA*_ = 0.01, **F.** RA with *A*_*RA*_ = 0.9.

To examine the dynamics of the surface colonization process and the mechanism that confers fitness advantage to PA over RA, we tracked the population partition to planktonic and biofilm sub-populations and the distribution of aggregate sizes of the biofilm sub-population. We first compared RA simulations with various attachment rates *A*_*RA*_ (Fig 4D–4F). Even with the highest *A*_*RA*_, where most of the cells were sessile, no large stress-protected aggregates formed (Fig 4F). This is in contrast with the optimal PA strategy that produced large stress-protected aggregates (Fig 4B). These large aggregates were established on day 1, though not reaching the size required for protection (i.e. Q_L_ = 40 cells) until day 2. Notably, during day 2 these aggregates grew massively and reached a size of hundreds of cells–way over the required protection-size–and persisted until the end of the simulation.

### The underlying mechanism that explains the fitness advantage of PA strategies

To understand how large aggregates form and persist under the optimal PA strategy, we analyzed the lineage composition of all aggregates. Aggregates that were enriched by attachments of planktonic cells are expected to be composed of many lineages, as opposed to clonally growing aggregates which are all descendants of a single founder cell. Large aggregates formed in PA simulations with the optimal Q_PA_ value are indeed composed of many lineages (Fig 4B), in contrast to the smaller unprotected aggregates formed by RA simulations, mostly composed of the founder cell progeny (Fig 4D–4F). An in-depth comparison between PA with the optimal attachment threshold (*Q*_*PA*_ = 12) and PA with a low *Q*_*PA*_ value (*Q*_*PA*_ = 4), revealed that under a low threshold more composite aggregates that did not reach protected density were generated. Some protected aggregates were formed, but they were much smaller and less stable than with the optimal *Q*_*PA*_. The total number of aggregates for *Q*_*PA*_ = 4 is higher, but since a smaller fraction of cells belongs to large protected aggregates, the total population yield is lower (Fig 4A). On the other hand, higher thresholds *Q*_*PA*_ = 20 did not produce protected aggregates at all (Fig 4C).

**Fig 5:**
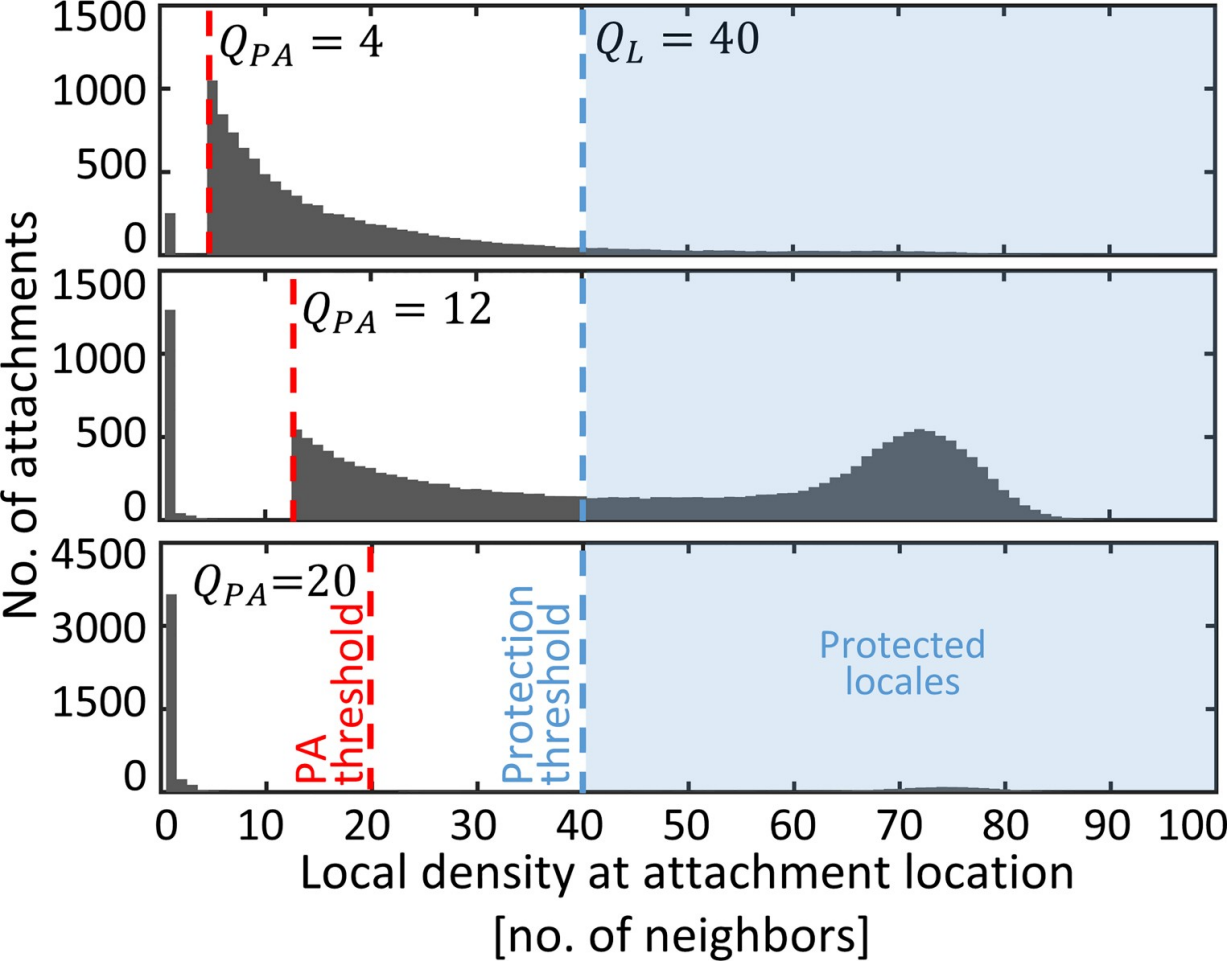
Attachments with the optimal PA (Q_PA_ = 12) enrich yet-to-be protected aggregates or maintain already-protected aggregates. Quantification of attachments of planktonic cells in various PA strategies, as a function of the local density at the attachment site. Histograms represent attachment events during the five day simulation (mean over 10 simulations). Standard errors are negligible in this scale.

We also analyzed the frequency of attachment events per local cell density (Fig 5). With RA most attachments are to vacant locales (0.79±0.01, 0.89±0.01, 0.99±0.01 mean±SE for high, balanced and low attachment rates, respectively). With low Q_PA_ most attachments are to very small aggregates and are thus inefficient in generating protected aggregates. PA facilitated the formation of protected aggregates, but their growth and contribution to survival are restricted without a sufficient flux of attaching cells. With optimal Q_PA_ attachments below Q_L_ are to larger and fewer aggregates, some of them eventually yield protection; and attachments above Q_L_ enrich already protected aggregates. With high Q_PA_ there are hardly any density-dependent attachments.

### PA confers fitness advantage over a range of environmental conditions

**Fig 6:**
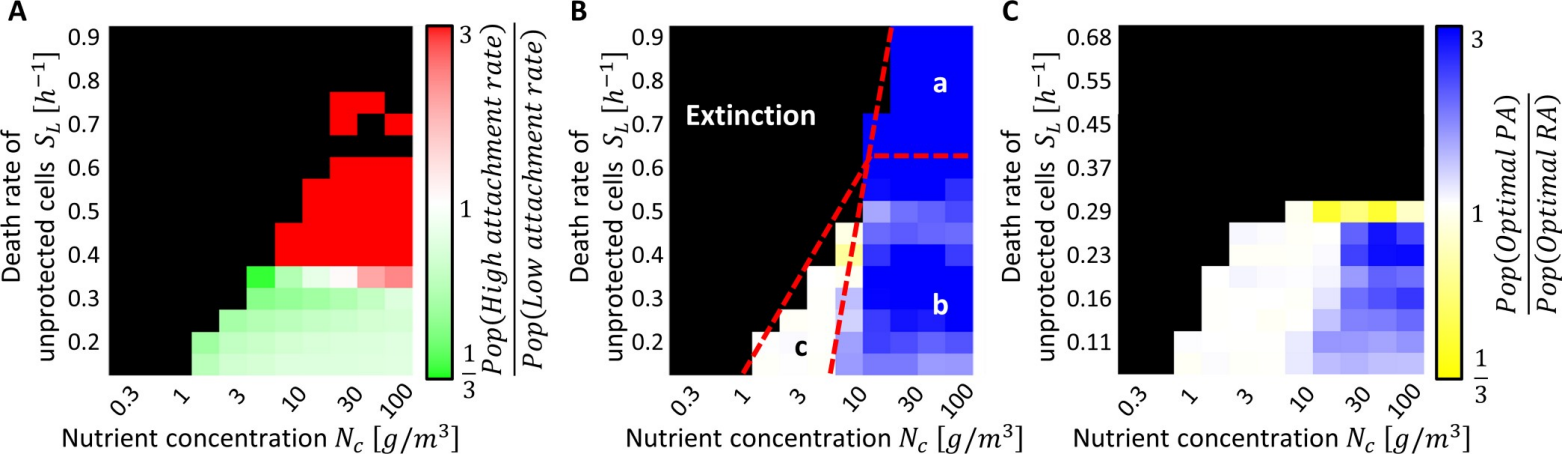
PA confers fitness advantage over RA in most of the analyzed phase plane and even extends the Hutchinsonian niche under periodic (but not constant) stress. **A.** Relative advantage of high-*A*_*RA*_ RA over low-*A*_*RA*_ RA under periodic stress, as a function of source nutrient concentration (N_c_) and death rate of unprotected cells (*S*_*L*_). Color bars represent the ratio between the population sizes at the end of a five day simulations. At low *S*_*L*_ values, the faster growth of the planktonic population compensated for the lower protection, and thus lower *A*_*RA*_ prevails (green zone). At higher *S*_*L*_ values, the formation of protected aggregates compensates for the lower growth rate, and higher *A*_*RA*_ rates result with a higher population size (red zone). **B.** Relative advantage of PA over RA under periodic stress. Zone a: The optimal PA strategy results with survival while all RA strategies lead to extinction in >80% of repeated simulations. Formation of protected aggregates occur only with PA. Zone b: PA is superior when growth rate is high enough to support the formation protected aggregates. Zone c: At Low N_c_ and low S_L_, PA and RA yield populations that are similar in size. **C.** Same as in (B) but under constant stress. The Y axis *S*_*L*_ values in (C) were modified so that the overall survival chances of unprotected cells during 24h of stress equal the survival chances during the 12h dry conditions of the periodic stress.

To examine the ecological relevance of the PA mechanism, and to find under what conditions PA confers fitness advantage, we performed a series of simulations that compared PA and RA at a range of nutrient concentrations (*N*_*c*_) and a range of death probabilities of unprotected cells (*S*_*L*_) (Fig 6). We first ran RA simulations with a range of attachment rate values (A_RA_). The emerging picture is described in Fig 6A: there is an area of the phase plane that led to extinction, and the rest of the plane is roughly partitioned into an area where lower attachment rates (preference for planktonic lifestyle, area in Green, Fig 6A) are superior, and an area where higher attachment rates (preference for biofilm lifestyle, area in Red, Fig 6A) are superior.

Next, we ran the same series of simulations, this time with PA with a range of Q_PA_ values, and compared the results of the optimal PA with the best RA strategy of the same point in the parameter space. We found that there exists a large area on the phase plane where PA is advantageous (area in Blue, Fig 6B). For a given S_L_, increasing values of *N*_*c*_ results in transitions in the outcome. At low *S*_*L*_ values (≤ 0.6 [*h*^−1^]), a first transition between extinction and survival occurs when *N*_*c*_ is high enough to replenish the planktonic sub-population after a dry period (Fig 6B, zone c). In zone c, PA and RA do equally well. A second transition occurs when *N*_*c*_ is high enough to enable the optimal PA to outperform any RA by enriching the nascent aggregates and granting them protection (Fig 6B, zone b). At high *S*_*L*_ values (> 0.6 [*h*^−1^]) unprotected survival is prohibited, thus high enough *N*_*c*_ values allow only PA strategies to survive (Fig 6B, zone a), thereby extending the Hutchinsonian niche [41] beyond the niche occupied by RA. Notably, in nearly all cases there exists some threshold Q_PA_ which can confer PA at least the same fitness as the best RA.

### Comparison of RA and PA strategies under constant stress

What happens if stress is constant and not periodic? To answer this question, we repeated the analysis under constant stress conditions. We found that the region of the phase plane where PA is better than RA is smaller and the mean advantage that PA confers is lower compared to periodic stress conditions: under constant stress, the most significant relative advantage of PA occurs where *N*_*c*_ ≥ 30[g/*m*^3^] and *S*_*L*_ ≤ 0.26[*h*^−1^], and the mean relative advantage of PA in this region is 1.8 ± 0.1; while under periodic stress, the mean relative advantage for the analogous conditions (i.e. equivalent survival chances for unprotected cells, *S*_*L*_ ≤ 0.45[*h*^−1^]) is 3 ± 0.25 (mean±SE) (Fig 6C). In addition, under stronger constant stress *S*_*L*_ ≥ 0.33[*h*^−1^], PA does not lead to survival and does not extend the Hutchinsonian niche of RA. This highlights the advantage of PA strategies over RA under periodic, rather than constant, stress.

### PA performance under different wet-dry durations

**Fig 7:**
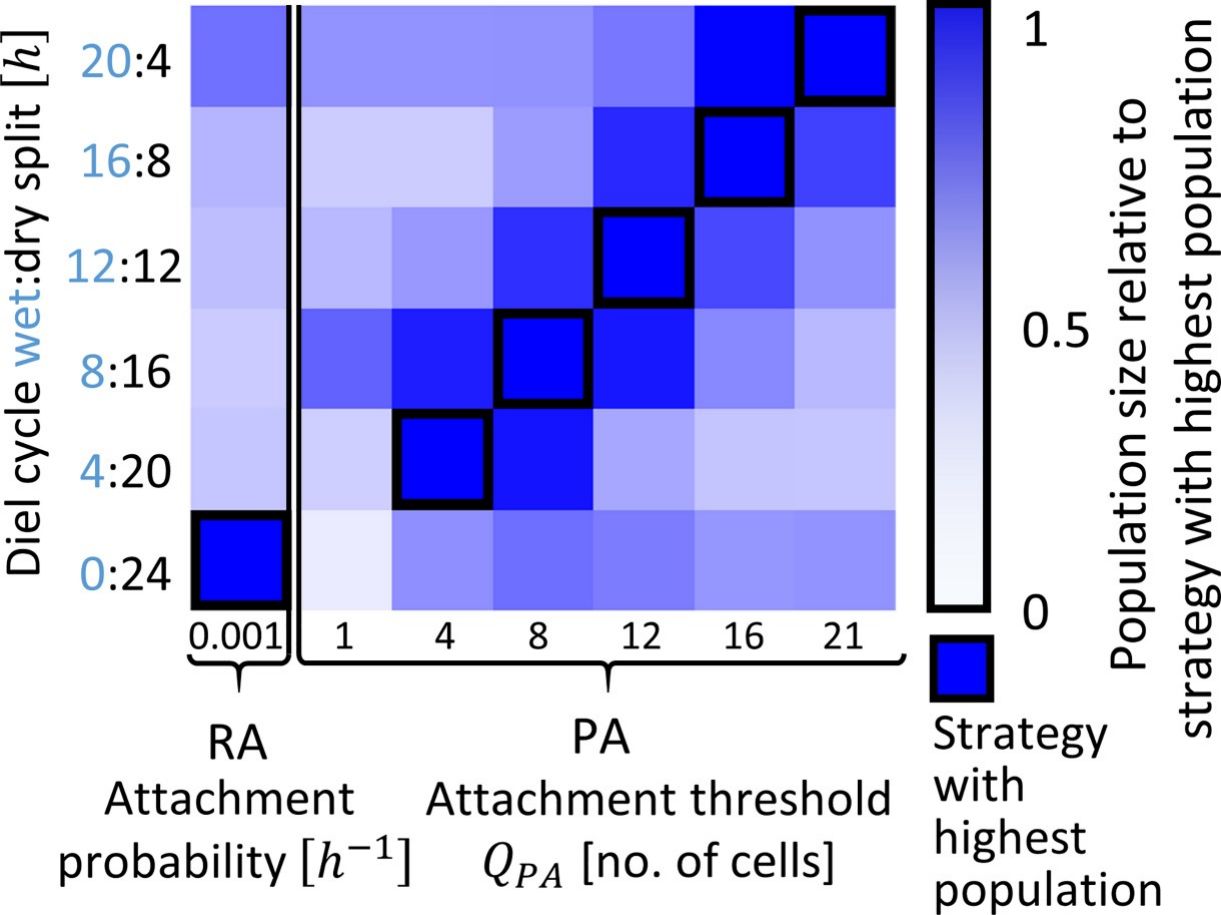
Comparison of different wet-dry diel cycle splits (duration of wet and dry periods), and their effects on the optimal strategy. The most successful strategy in each set of conditions is marked by a square, and the color of other strategies show their yield relative to the optimal one. Data is based on the mean value of 10 simulations.

Last, we asked how changes in the duration of the wet and dry periods over the diel cycle affect our results. We found that, under the studied conditions, PA confers advantage over all range of wet period lengths (4h to 20h per day). Moreover, we find that the optimal Q_PA_ changes with the length of the wet period: the longer the wet period the higher is the optimal Q_PA_ (Fig 7). This is because as the duration of the wet period increases, larger aggregates are formed clonally, and a higher Q_PA_ allocates less planktonic cells to the aggregates that will likely not reach the protected size.

## Discussion

In this work we used individual-based modeling to study early bacterial surface colonization under periodic stress. Importantly, we reflected in our simulations the tradeoff between growth and survival of planktonic and biofilm lifestyles. We show how surface-attachment strategies of individual cells influence self-organization during colonization on a surface and most importantly how this in turn impacts fitness. We find that across a wide range of conditions under periodic stress, a simple strategy, such as preferential attachment (PA) to existing aggregates above a given size, carries a large fitness advantage over any random attachment (RA) strategy. The improvement in fitness is achieved by a more optimal partition between the planktonic and biofilm sub-populations and precise modulation of the aggregate-size distribution dynamics, both controlled by the attachment decisions of individual-cells.

**Fig 8:**
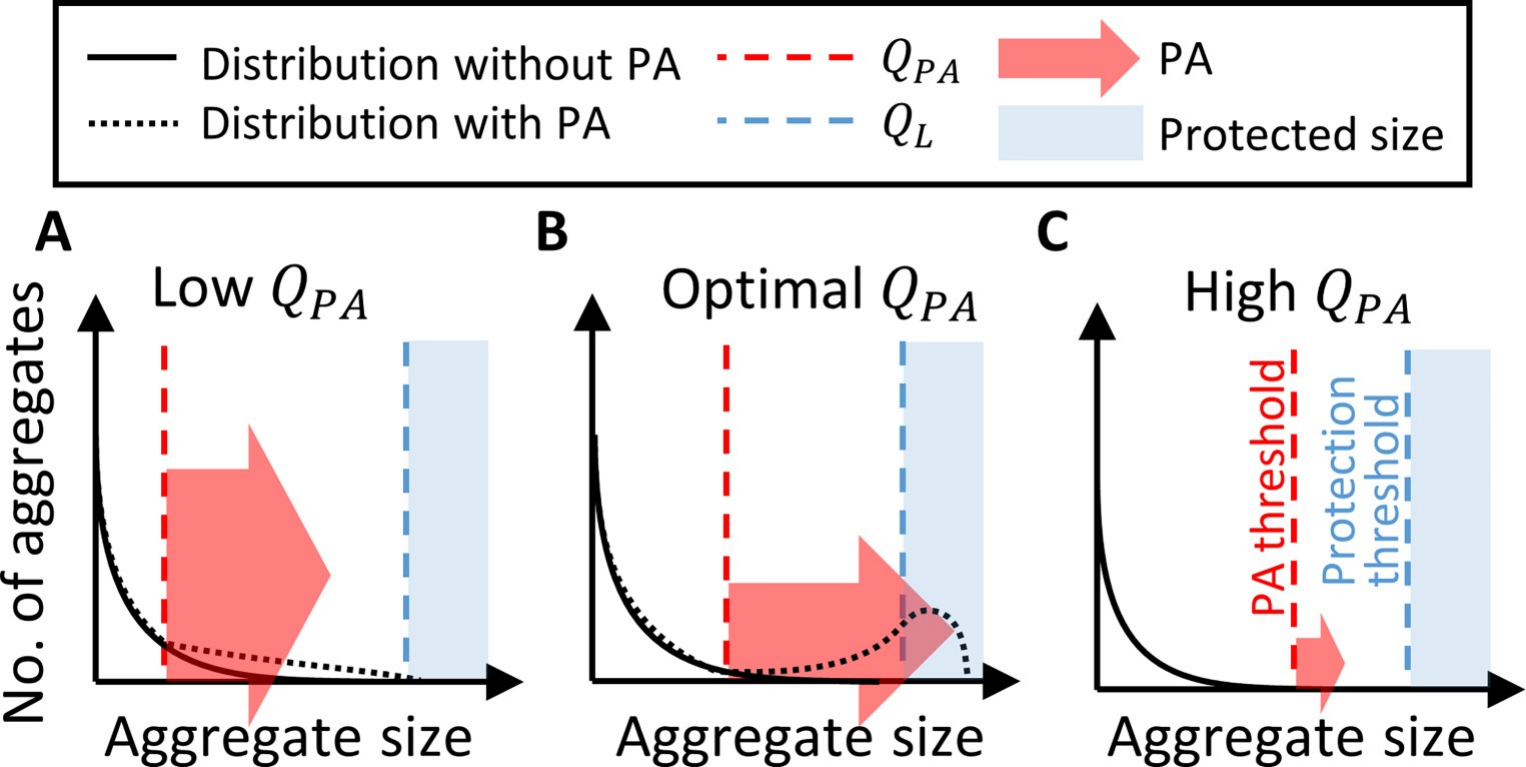
The underlying mechanism of the PA strategy. The beneficial effect of preferential attachment is strongly dependent on the attachment density threshold. PA accelerates the growth of larger aggregates by recruitment of planktonic bacteria. The effect of the PA mechanism depends on the attachment density threshold, **A.** Low attachment density threshold: Most enriched aggregates do not surpass the protected zone, since the total rate of preferential attachments is divided among a large number of aggregates. **B.** Optimal attachment density threshold: Preferential attachment to a small group of aggregates which surpass the critical size. **C.** High attachment density threshold: (few or) no aggregates are large enough to draw the planktonic population.

The early phases of bacterial surface colonization have a crucial spatio-temporal aspect. When only a small fraction of the surface is colonized and cells are not uniformly distributed over the surface, the time and location of surface-attachment of planktonic cells affects the fitness and survivability of the individual cell, as well as the nearby population. PA modulates the aggregate-size distribution via the attachment threshold *Q*_*PA*_. The allocation of planktonic cell attachments to aggregate growth can be optimized by fine-tuning the *Q*_*PA*_. The optimal *Q*_*PA*_ results with the largest part of the population protected from stress by carrying aggregates beyond the critical size (Fig 8).

The temporal periodicity of stress plays a major role in the relative advantage of PA over RA. Under constant stress, environments harsh enough to attenuate aggregate growth throughout the colonization process will subsequently render PA ineffective. Without the temporal variation of stress, PA does not extend the Hutchinsonian niche of the system.

It is important to note that PA is not expected to be beneficial under all conditions and all combinations of variables and parameters. The parameter space in our model is huge and it is unfeasible to thoroughly scan it. We demonstrate that under some reasonable assumptions and environmentally relevant conditions, PA can provide fitness advantage over RA.

Generally, PA can be a result of both passive and active mechanisms. An example of a passive mechanism is the reported biased surface-movement toward dense areas controlled by the sticky trails of polysaccharide [4]. Active mechanisms may involve chemotactic movement toward aggregates [42] or informed attachment decisions like the one we modeled here. Nevertheless, other–both passive and active–mechanisms can equally well lead to a PA process where aggregates are enriched in a rich-get-richer manner and thus may confer fitness advantage in environments that select for collective protection.

In our model we assumed that information on local cell density is available to foraging planktonic cells. There are several possible mechanisms that can facilitate the attachment of planktonic cells to denser locations. Quorum-sensing (QS) signals in early surface colonization on natural unsaturated surfaces such as plant root and leaf surfaces are highly localized, and quorum size can be surprisingly small: even as low as 10 cells [43, 44]. Other sensing systems, such as peptidoglycan sensing by bacteria, can serve as indicators of cell proximity [45]. Adherence to Psl trails [4] is another AEM that increases the probability of cells’ irreversible attachment to existing aggregates, biased towards larger established aggregates.

In this study we considered homogenous populations (i.e. all cells are from a single species and employ the same strategy) and we measured fitness by population yield. This allowed us to quantify the effect that individual-cell behavior has on the collective fitness, that would have been disrupted (because of interactions between competing strategies) if we chose to rely on evaluating fitness based on competition simulations that are commonly used to estimate relative fitness and to study evolutionary dynamics [46]. Preliminary results of competition simulations between populations that employ different PA or RA strategies showed various interesting dynamics, including commonly known evolutionary scenarios such as co-existence, cooperation, exploitation, cheating, and invasion. For example, populations with the optimal PA strategy can be exploited by invaders with non-optimal higher PA threshold, that can join established protected aggregates later and benefit form a longer planktonic phase at a higher growth rate. The protected aggregates of the optimal PA strategy have a lower relatedness in comparison to the mostly clonal aggregates created by RA and high threshold PA strategies (Fig 4 and S2 Fig), and thus PA is not expected to be an Evolutionary Stable Strategy [47, 48]. These intriguing initial results demonstrate the richness of the studied system and open the way for further research which was beyond the scope of the current study.

The present study demonstrates the impact that PA may have on bacterial fitness during early surface colonization, particularly under periodic stress. The general principles of the model are applicable to different stresses such as desiccation, antibiotics and predation. Our modeling results, together with evidence for PA processes in bacterial surface colonization in both experimental and natural systems [4, 5], call for further study of PA and similar processes and their contribution to collective protection in microbial ecosystems.

## Methods

Our Individual Based Model is based on the Repast Platform [49]. The simulation domain is a two-dimensional 1mm by 1mm square that is comprised of a bulk liquid phase and a surface phase. The individuals are bacterial cells, either planktonic or sessile. Planktonic cells move at random. Sessile bacteria are static and move only by physical shoving to avoid overlaps, with aggregates consisting of a single layer of cells. A single nutrient resource is consumed by the cells, and replenished by diffusion into the domain from an infinite source. The cells’ growth rate, actions, and death probability are determined by their state and environmental conditions, i.e. nutrient concentration, stress, and local surface density, as described in Tables 1 and 2. All simulations begin with 100 planktonic cells, and a nutrient concentration in equilibrium with the source. For a detailed description of the model, see S1 Text.

## Supporting information

S1 Text Detailed model description. (DOCX)

S1 Video Side-by-side view of two representative simulations. RA strategy with high attachment rate (A_RA_ = 0.9 [h^-1^]) (left) and the optimal PA strategy (Q_PA_ = 12) (right).
(AVI)

S1 Fig Population sizes at the end of a five diel cycle simulations with different hill coefficients of the attachment probability function. Each data point is mean±SE of 10 simulations. The dashed line represents the population size of the optimal RA simulation, among all tested *A*_*RA*_ values. Inset: PA probability function per time step. Dot color matches the probability function shown in the inset.
(TIF)

S2 Fig Relatedness within protected aggregates depends on cells’ strategy and external conditions. Relatedness is computed as the mean pairwise relatedness of all cell pairs (defined arbitrarily as 1 for cells from the same lineage and 0 otherwise) within each aggregate, averaged over all aggregates with 100 or more cells. Plot shows mean±SE of 10 simulations. (A) Relatedness as a function of wet interval duration within the diel cycle. (B) Relatedness as a function of Q_PA_ value, at 12:12 hours wet-dry diel cycle.
(TIF)
