# Supplementary material for: Bacterial surface colonization, preferential attachment and fitness under periodic stress
Source: PLoS Comput Biol. 2019 Mar 5;15(3):e1006815. doi: 10.1371/journal.pcbi.1006815 (PMC6420035; doi:10.1371/journal.pcbi.1006815)
Supplement: S1 Text — (DOCX) [file pcbi.1006815.s001.docx]

## **Supplementary Information Text**

## **Model overview**

Our Individual Based Model, based on the Repast Platform [1], simulates the early bacterial surface colonization process. The description of the model follows ODD, the standard protocol for Individual Based Model (IBM) description[2]. An IBM is the most appropriate approach to study the emergent properties of the system, including self-organization and fitness, resulting from individual behavior [3]. In our case, the spatio-temporal organization of bacteria on surfaces emerges from the properties and behavior of individual bacterial cells. The model evaluates the fitness of populations by means of population size (or yield), and allows to assess the fitness advantage conferred by employing various attachment strategies. In addition, it provides a wealth of information, inaccessible in experimental approaches, that shed light on the underlying mechanism of superior strategies. The source code for the model is available at <https://github.com/MaorG/leaf04>.

### **Model description, entities, state variables and scales**

The simulation domain is a two-dimensional 1mm by 1mm square. The domain is comprised of two phases – bulk liquid and a surface. The conditions within the simulation are periodic, changing from “wet” to “dry” conditions at regular intervals within 24-hour cycles.

The conditions are set for each time step using the following function

$$\left( 1 \right) W\left( t \right)=\left\{ \begin{matrix} 1 & \text{mod}\left( t,24 \right)\leq H \\ 0 & \text{mod}\left( t,24 \right)>H \end{matrix} \right.$$

Where $H$ is the duration of the wet period.

A single nutrient resource is consumed by the individuals (i.e. agents), and replenished by permeation into the domain, in agreement with existing phyllosphere-related simulations [4]. The individuals are round cells with a radius that can vary between 1-2 μm, and can take one of two possible states: planktonic or sessile (i.e. surface-attached). Planktonic cells inhabit the liquid phase and move at random. Sessile bacteria are located on the surface phase, with aggregates consisting of a planar layer of non-overlapping cells. The simulation is performed in uniform time steps of 0.05 hours (3 minutes).

### **Process overview**

Bacterial processes: Cells can switch between planktonic and sessile states (and vice versa) at random:

$$\left( 2 \right) A_{i, random}(x,t)=A_{RA}$$

$${\left( 3 \right) D}_{i, random}(x,t)=D$$

Additionally, a planktonic cell may sense the local number of sessile cells on the surface phase within a neighborhood of 10μm radius ($Q_{i}(x,t)$) and attach at probabilities dependent on that local cell density, with a hill equation as the response function:

$$\left( 4 \right) A_{i, preferential}\left( x,t \right)={A^{'}}_{PA}\cdot\frac{1}{\left( \frac{Q_{PA}}{Q_{i}\left( x,t \right)} \right)^{n}+1}$$

Since random attachment is also possible, we add (2) and (4) and get

$$\left( 5 \right) A_{i}\left( x,t \right)={A^{'}}_{PA}\cdot\frac{1}{\left( \frac{Q_{PA}}{Q_{i}\left( x,t \right)} \right)^{n}+1}+A_{RA}$$

and we set ${A^{'}}_{PA}=A_{PA}-A_{RA}$ to account for the random probability to attach at $Q_{i}\left( x,t \right)<Q_{PA}$, resulting with

$$\left( 6 \right) A_{i}\left( x,t \right)={(A}_{PA}-A_{RA})\cdot\frac{1}{\left( \frac{Q_{PA}}{Q_{i}\left( x,t \right)} \right)^{n}+1}+A_{RA}$$

For all of the simulations used in the main text, we set $n=1000$, so that this function is practically a step function with $A_{RA}$ probability of preferential attachment when$Q_{i}\left( x,t \right)<Q_{PA}$ and ${A^{'}}_{PA}$ probability otherwise. Lower values of $n$, which generate a more gradual response function, were tested and did not provide a significant advantage over $n=1000$ (Fig S1).

Stress is modeled as a probability function of the local cell density, namely the number of cells on the surface in a 10μm radius neighborhood. Planktonic cells are considered to have local density of 0 for stress calculations. At low densities (below Q_L_, see Fig. 1E and Tables 1 and 2 in the main text), cells experience desiccation stress. At high densities (above Q_H_), found typically at the centers of aggregates, cells experience stress that mimics nutrient depletion and toxins buildup. Therefore, the probability function of cell death is composed of three constant functions at different intervals:

- At intermediate densities, $Q_{L}\leq Q\left( x_{i},t \right)<Q_{H}$, death probability is low ($S_{B})$.
- At low densities $Q\left( x_{i},t \right)<Q_{L}$, death probability depends on the external conditions ($S_{L}{+S}_{B}$ when dry, and $S_{B}$ when wet).
- At high densities $Q\left( x_{i},t \right)\geq Q_{H}$, cell death probability is constant, $S_{H}+S_{B}$.

Put together, we get:

$$\left( 7 \right) S_{i}\left( x_{i},t \right)= \left\{ \begin{matrix} (1-W\left( t \right))\cdot S_{L}+S_{B} & Q\left( x_{i},t \right)<Q_{L} \\ S_{H}+S_{B} & Q\left( x_{i},t \right)\geq Q_{H} \\ S_{B} & \text{otherwise} \end{matrix} \right.$$

Where $W\left( t \right)$denotes the current wetness as above.

Bacterial growth follows Monod kinetics with $\mu_{max}=0.4h^{-1}$ and $K_{s}=0.3 g{\cdot m}^{-3}$ for planktonic and sessile bacteria, but sessile bacteria invest half the gain in biomass to EPS production (Tables 1 and 2). Therefore, growth rate for a single cell is

$$\left( 8 \right) \mu=\mu_{max} \frac{c}{c+K_{s}}$$

where c is nutrient concentration. At each time step, each cell consumes nutrients and grows in mass, so planktonic cells’ mass gain over the span of a time step $\Delta t$ is in the form of exponential growth with a constant rate:

$$\left( 9 \right) {\Delta m}_{i}\left( t+\Delta t \right)=m_{i}\left( t \right)\cdot\exp\left( \mu\Delta t \right)-m_{i}\left( t \right)$$

Since sessile cells invest $f$ fraction of their biomass gain in EPS production, their mass gain is reduced accordingly:

$$\left( 10 \right) \Delta m_{i}\left( t+\Delta t \right)=\left( 1-f \right)\cdot\left( m_{i}\left( t \right)\cdot\exp\left( \mu\Delta t \right)-m_{i}\left( t \right) \right)$$

And thus, for cell *i*:

$$\left( 11 \right) m_{i}\left( t+\Delta t \right)=\left( 1-f_{i} \right)\cdot m_{i}\left( t \right)\cdot\exp\left( \mu\Delta t \right)+f_{i}m_{i}\left( t \right)$$

Where $f_{i}$ is 0.5 when the i-th cell is sessile, and 0 when it is planktonic.

When the cell’s mass surpasses some critical value it divides into two cells each with half the mass.

As sessile cells on the surface grow and divide, or when planktonic cells change their state to sessile, they may spatially overlap on the surface. In such cases they exert lateral elastic force and move away from each other.

Simulation domain processes: The hydration conditions are updated at each time step, changing from ‘wet’ to ‘dry’ and back at regular intervals. The nutrient flow into the domain is modelled as a uniform diffusion, proportional to the concentration difference between the concentration in the model and a reservoir of constant concentration. Diffusion of the nutrient within the domain is modeled as global mean concentration in the grid-cells, since the diffusion coefficient of sugars in water is large enough to be treated as instant. The nutrient consumed by the bacterial cells is subtracted from the overall quantity of nutrients in the domain at each time-step. When the projected consumption of nutrients over the next time-step in a grid-cell surpasses its content, the consumption is restricted so that concentration will not drop below zero.

### **Design concepts**

Emergence: The mechanisms of self-organization are introduced by cell decisions, and therefore any observed system-level spatial phenomenon that deviates from a null model emerges from the individual traits.

Sensing: Cells are affected by the local cell density in a 10μm radius environment around them. Planktonic cells attachment rate may depend on local density of sessile cells, and sessile cells are affected by death rate, which depends on local density.

Adaptation: Individual cells may change their state from planktonic to sessile either at uniformly random rate, or with dependence on the local density of sessile cells on the surface.

Interaction: physical shoving through elastic forces. individual cell’s attachment decisions can be affected by nearby cells. Cells’ spatial organization on the surface affects their stress resistance.

Stochasticity: Division mass: values are drawn from normal distribution. Death by stress: probability function of environmental variables and local density. Attachment / detachment: probability functions portraying the stochastic nature of the decision of an individual cell to attach to or detach from the surface.

Observations: Total population size and partition to states, total and local nutrient concentration, individual-cell properties (state, location, size, number of neighbors, growth rate, lineage history), history log of individual-cells' actions and responses (division, death, attachment, detachment).

Initialization: 100 individual cells in the planktonic state. The initial concentration of nutrients is identical to the concentration of the reservoir.

### **Simulated scenarios**

We scanned the parameter space of nutrient concentrations, stress response functions, preferential attachment thresholds and rates and random attachment rates, partitions of the diel cycle into wet/dry intervals.

### **Input**

The environmental conditions are imposed as state variables, changing from “wet” to “dry” at 12h intervals.

Domain properties are configurable, as are the properties of the cells.

**References (SM)**

1. Collier N. Repast: An extensible framework for agent simulation. The University of Chicago’s Social Science Research. 2003;36:2003.

2. Grimm V, Berger U, Bastiansen F, Eliassen S, Ginot V, Giske J, et al. A standard protocol for describing individual-based and agent-based models. Ecological Modelling. 2006;198(1):115-26. doi: <https://doi.org/10.1016/j.ecolmodel.2006.04.023>.

3. Grimm V, Railsback SF. Individual-based Modeling and Ecology:(Princeton Series in Theoretical and Computational Biology). 2005.

4. Van Der Wal A, Tecon R, Kreft J-U, Mooij WM, Leveau JH. Explaining bacterial dispersion on leaf surfaces with an individual-based model (PHYLLOSIM). PloS one. 2013;8(10):e75633. Epub 2013/10/15. doi: 10.1371/journal.pone.0075633. PubMed PMID: 24124501; PubMed Central PMCID: PMCPMC3790818.
