# Supplementary figures and images for: Bacterial surface colonization, preferential attachment and fitness under periodic stress
Source: PLoS Comput Biol. 2019 Mar 5;15(3):e1006815. doi: 10.1371/journal.pcbi.1006815 (PMC6420035; doi:10.1371/journal.pcbi.1006815)

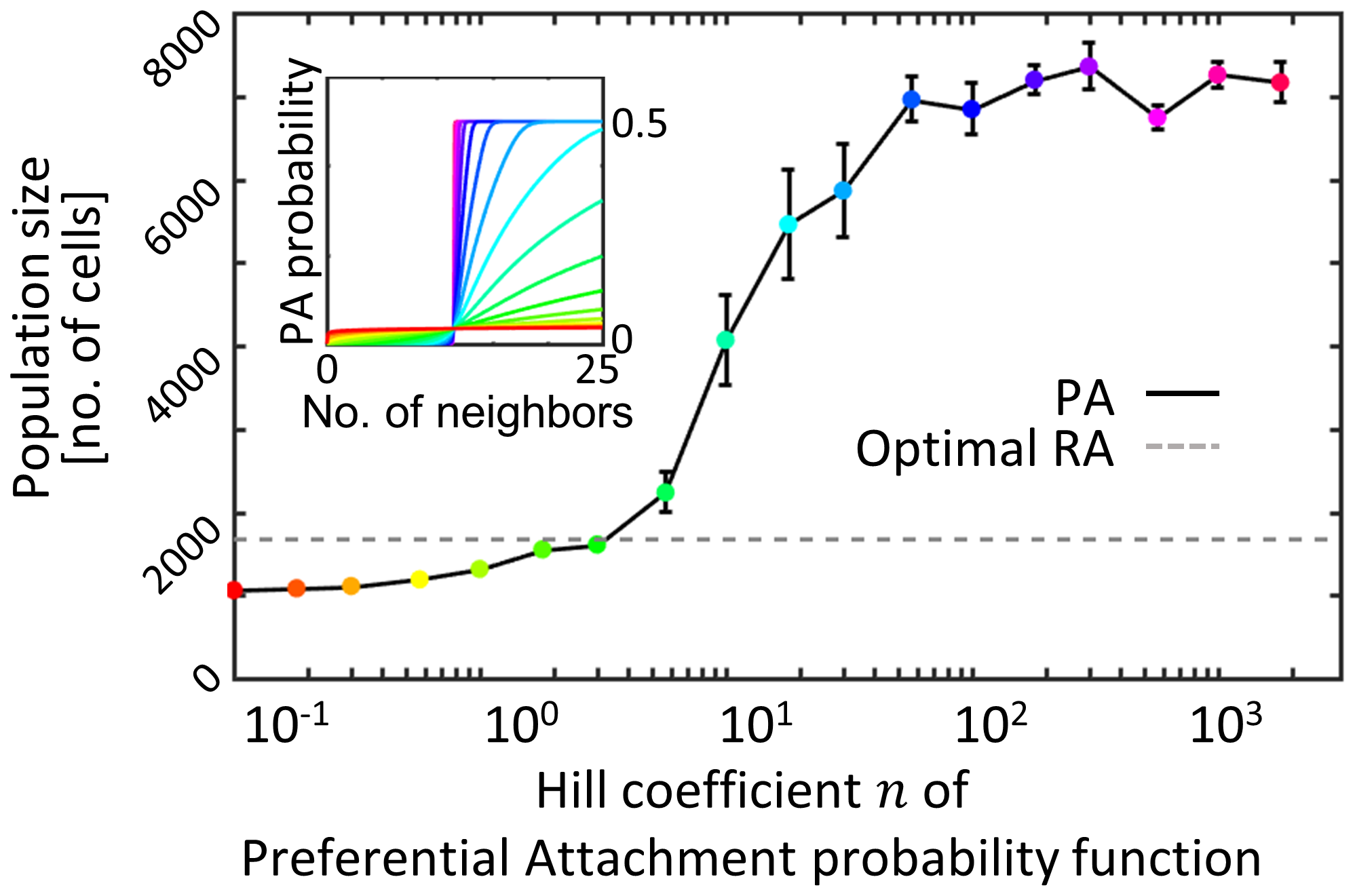

Supplement: S1 Fig — Each data point is mean±SE of 10 simulations. The dashed line represents the population size of the optimal RA simulation, among all tested ARA values. Inset: PA probability function per time step. Dot color matches the probability function shown in the inset. (TIF) [file pcbi.1006815.s003.tif]

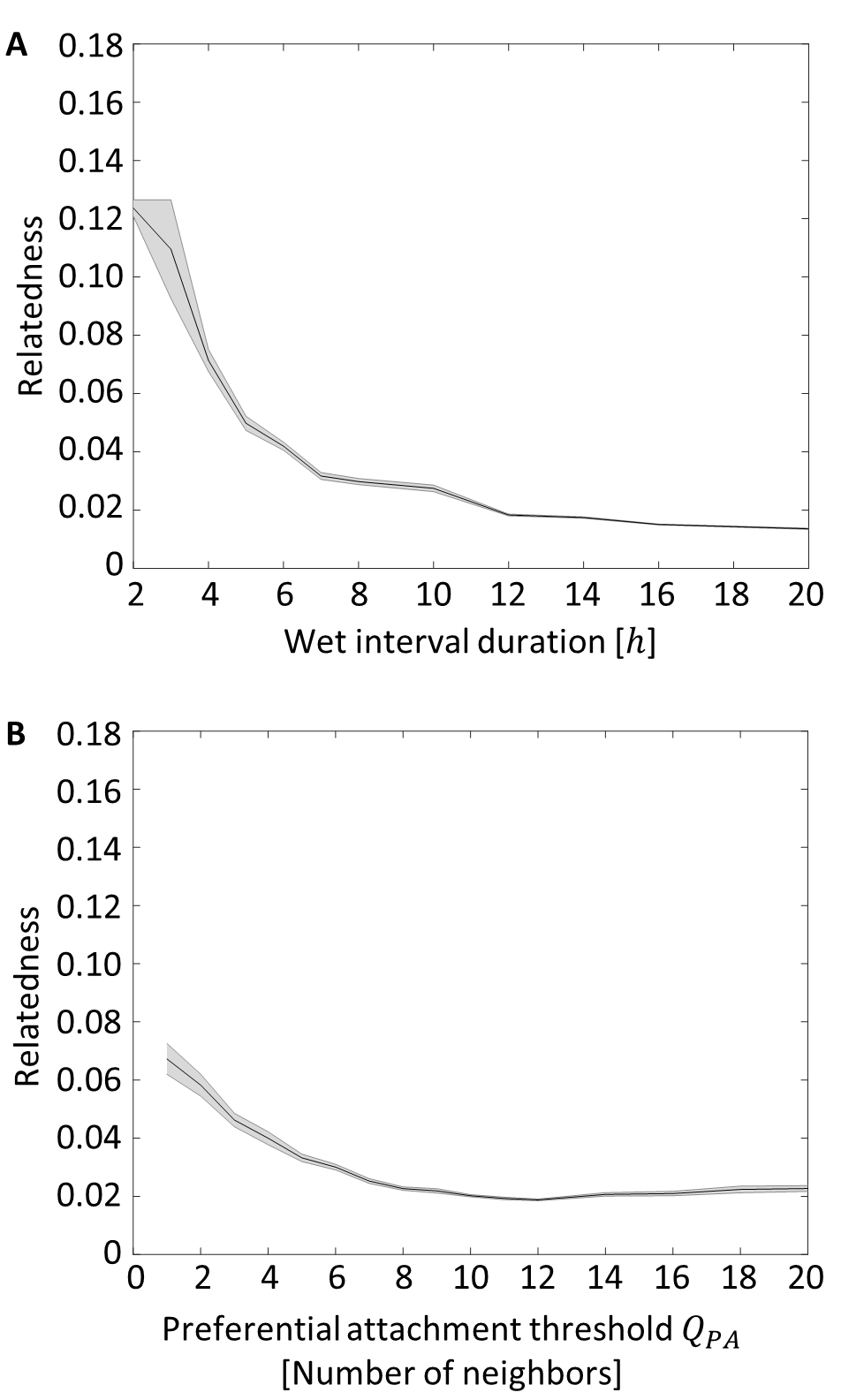

Supplement: S2 Fig — Relatedness is computed as the mean pairwise relatedness of all cell pairs (defined arbitrarily as 1 for cells from the same lineage and 0 otherwise) within each aggregate, averaged over all aggregates with 100 or more cells. Plot shows mean±SE of 10 simulations. (A) Relatedness as a function of wet interval duration within the diel cycle. (B) Relatedness as a function of QPA value, at 12:12 hours wet-dry diel cycle. (TIF) [file pcbi.1006815.s004.tif]
